# Experiences, acceptability and feasibility of an isometric exercise intervention for stage 1 hypertension: embedded qualitative study in a randomised controlled feasibility trial

**DOI:** 10.1186/s40814-024-01539-8

**Published:** 2024-08-26

**Authors:** Melanie Rees-Roberts, Rachel Borthwick, Ellie Santer, John Darby, Alan West, Jamie M. O’Driscoll, Tracy Pellatt-Higgins, Katerina Gousia, Vanessa Short, Tim Doulton, Jim Wiles, Chris Farmer, Douglas MacInnes

**Affiliations:** 1 Centre for Health Services Studies, University of Kent, Canterbury, UK; 2 Public co-applicant, Kent, UK; 3 School of Psychology and Life Sciences, Canterbury Christ Church University, Canterbury, Kent UK; 4 East Kent Hospitals University NHS Foundation Trust, Canterbury, UK; 5 School of Nursing, Midwifery and Social Work, Canterbury Christ Church University, Canterbury, Kent UK

**Keywords:** Exercise, Isometric, Hypertension, Primary care, UK National Health Service, Qualitative research

## Abstract

**Background:**

Healthy lifestyle changes for patients with stage 1 hypertension are recommended before antihypertensive medication. Exercise has antihypertensive benefits; however, low adoption and high attrition are common. Patients need easily adoptable, effective and manageable exercise interventions that can be sustained for life. We present participant and stakeholder perceptions of an isometric exercise intervention for stage 1 hypertension delivered in the National Health Service (NHS, UK).

**Methods:**

An embedded qualitative study within a randomised-controlled feasibility study included intervention arm participants (*n* = 10), healthcare professionals from participating NHS sites (*n* = 3) and non-participating NHS sites (*n* = 5) taking part in semi-structured interviews to explore feasibility of delivering an isometric exercise intervention within the study design and an NHS context. Data was analysed using reflective thematic analysis.

**Results:**

Three themes were identified: study deliverability; motivators and barriers; support for study participation. Findings indicated that the study was well designed. Health benefits, unwillingness to take medication, altruism and interest in the study helped motivation and adherence. Study support received was good, but healthcare professionals were insecure in intervention delivery with regular training/supervision needed. Perception of health improvement was mixed, but, in some, uptake of wider lifestyle changes resulted from participation. Stakeholders felt that current service challenges/demand would make implementation challenging.

**Conclusions:**

Despite participant positivity, delivery of an isometric intervention in an NHS setting was considered challenging given the current service demand, although possible with robust effectiveness evidence. Findings support further effectiveness data and implementation development of the isometric exercise intervention.

**Trial registration:**

ISRCTN, ISRCTN13472393. Registered 18 September 2020.

**Supplementary Information:**

The online version contains supplementary material available at 10.1186/s40814-024-01539-8.

## Key messages regarding feasibility

- **What uncertainties existed regarding the feasibility?**
  There are no other published randomised controlled trials of isometric wall squat for the treatment of hypertension in the NHS, despite laboratory-based studies indicating efficacy and secondary data analysis indicating superiority over other forms of isometric of exercise and other types of exercise programme. Therefore, feasibility studies are important in this underdeveloped field and where exercise interventions can be difficult to conduct due to no options for placebo control and due to attrition and adherence problems. Furthermore, it is unclear how exercise interventions can be implemented in an NHS context.
- **What are the key feasibility findings?**
  Although healthcare professionals were able to deliver the intervention, the evidence and feedback received through this embedded qualitative study has highlighted key barriers to uptake in standard practice including healthcare professionals’ insecurity in intervention delivery. Participants found the intervention acceptable and key ingredients for adherence identified. Flexibility is key to enable participants to adapt and conduct the exercises within their comfort zone. Motivation to take part included the health benefits, unwillingness to take medication, altruism and interest in the study. Support received was good, and despite perception of health improvement being mixed, in some, participation encouraged uptake of wider lifestyle changes.
- **What are the implications of the feasibility findings for the design of the main study?**
  Feasibility work has informed the development of a follow-on effectiveness study with simplified protocols for self-delivery of the personalised wall squat programme and reduced need for equipment. Changes to inclusion/exclusion criteria to reduce screen failure and improved recruitment strategies have been identified. Refinements to the study documentation and procedures were also identified for full trial.

## Introduction

High blood pressure or hypertension is diagnosed in stages according to the severity. Early hypertension (stage 1) is diagnosed upon clinical evidence of blood pressure above 140mmgHg systolic over 90 mmHg diastolic (140/90 mmHg) or a home blood pressure measurement average of ≥ 135/85 mmHg [1]. In 2019, hypertension was the leading global cause of mortality and morbidity after tobacco use [2]. In 2021, thirty percent (30%) of adults living in England were known to have hypertension, including 15% remaining untreated [3]. Differences in prevalence of hypertension across populations is linked to key risk factors including age, sex, ethnicity, genetic factors, social deprivation, co-existing conditions, lifestyle and mental health factors [4]. Tackling hypertension is one of the top priorities outlined by the National Health Service (NHS) in the Core20 plus 5 health strategy for reducing health inequalities across England [5].

Hypertension is a modifiable risk factor and linearly associated with an escalated risk of cardiovascular disease and other serious conditions such as stroke, heart attack and kidney disease [6]. Guidance for treatment of adults with stage 1 hypertension (grade 1 in European guidelines) is multifaceted including lifestyle advice and then anti-hypertensive medication if blood pressure remains elevated [1, 7]. The goal of antihypertensive therapy is to reduce resting blood pressure to within recommended ranges for age and co-morbidities [8]. Globally, control rates amongst people with hypertension on treatment tend to be extremely low at around 23% for women and 18% for men [9]. In the UK, approximately 50% of people clinically diagnosed with any stage of hypertension and on treatment fail to achieve optimal blood pressure control, largely resulting from non-adherence to prescribed medication due to undesirable side effects [9, 10]. Furthermore, evidence suggests up to 32% of patients with stage 1 hypertension progress to stage 2 hypertension, indicating failure to manage hypertension during earlier stages [11].

A reduction of only 10 mmHg in systolic blood pressure is associated with a 17% reduction in coronary heart disease risk, 27% reduction in stroke risk, 28% reduction in heart failure risk and 13% reduction in all-cause mortality [12]. Therefore, there is a vital need for alternative antihypertensive treatments that may be more acceptable and without common side effects. Lifestyle options (smoking cessation/alcohol use/diet/exercise/weight loss) should not be overlooked, as they can be as effective as medication in achieving this effect [13, 14]. Current UK exercise guidelines for stage 1 hypertension are the same as healthy individuals: at least 150 min of moderate intensity aerobic exercise per week [15]. Adoption of recommended levels of physical activity is a significant barrier in managing blood pressure [16, 17] as around 39% of UK adults (approximately 20 million people) fail to meet these recommendations [18, 19]. Although exercise is proven to have antihypertensive effects [12, 20], in some studies, aerobic exercise programmes have failed to reduce blood pressure, likely due to poor adherence and attrition where high amounts of aerobic exercise are required [21–23]. Therefore, alternative, more achievable exercise regimens are required to complement current approaches [23].

A recent meta-analysis [14] has indicated that a certain type of exercise, called isometric exercise, results in larger reductions in blood pressure compared to traditional aerobic exercise and combination aerobic/resistance training [13, 14]. Isometric exercise involves holding a fixed body position for a short period of time, for example a stationary wall squat, or hand grip exercise. Of the three specific isometric exercises evaluated to date in randomised controlled trials for blood pressure modulation (wall squat, leg extension and hand grip), the wall squat consistently produced the largest reductions in both systolic and diastolic blood pressure of adults studied in excess of 8/4 mmHg [14]. Isometric exercise offers advantages over aerobic exercise as it is completed in a much shorter timeframe [13, 24] and can be conducted at home without any equipment which increases the likelihood of good adherence, making it an attractive lifestyle option for hypertension [16, 22]. A study of isometric exercise wall squats over 1 year, conducted at home after an initial exercise laboratory visit, demonstrated impressive long-term adherence [25].

Most isometric exercise trials to date involve delivery of optimised programmes by sports/exercise experts within exercise laboratory settings including across normotensive [26, 27], pre-hypertensive [28, 29] and hypertensive populations [30, 31] producing positive effects of isometric exercise on blood pressure [14]. To understand the true potential of isometric exercise to the general hypertensive population, studies of delivery in healthcare settings are required. This paper explores deliverability and feasibility of a wall squat isometric exercise programme for hypertension in the NHS.

## Methods

### Study design

**Fig. 1:**
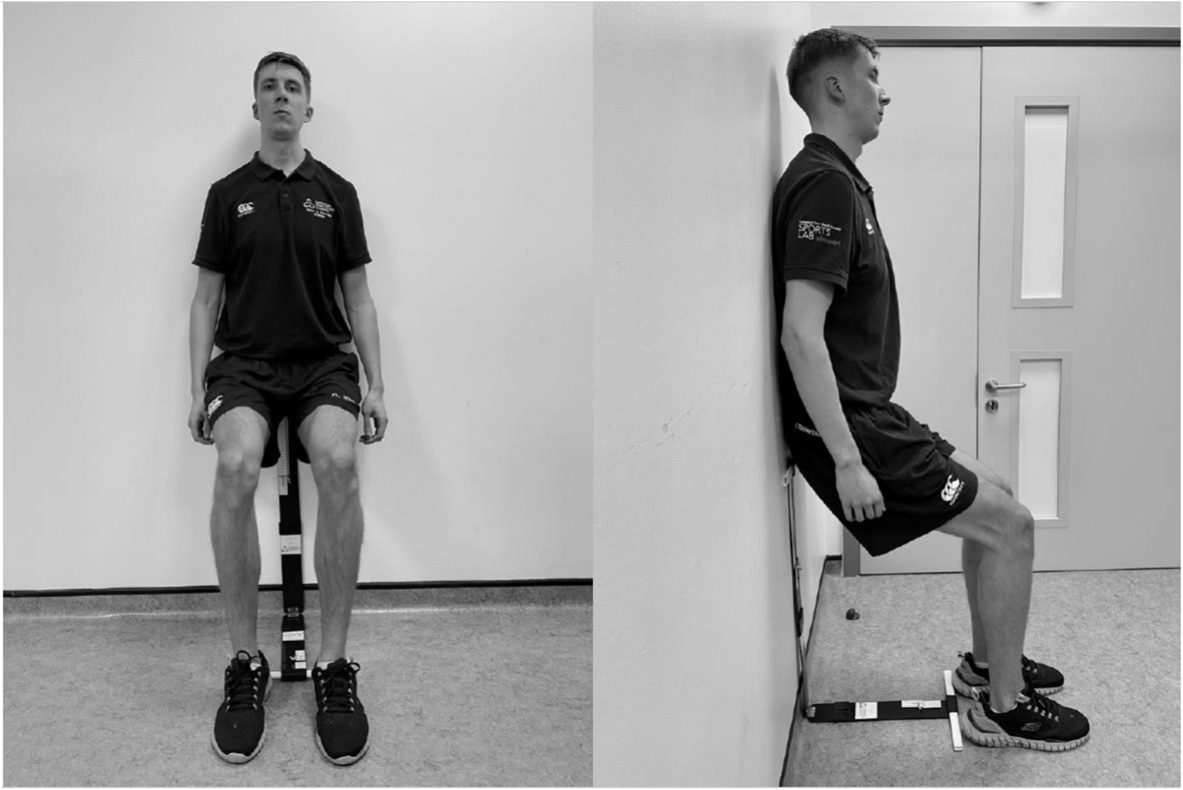
Isometric exercise intervention wall squat

An embedded qualitative study was conducted as part of a multi-centre randomised controlled feasibility study conducted within NHS settings (primary care, secondary care and in pharmacy settings) in southeast England [32]. In summary, the randomised controlled trial consisted of a parallel group design, randomising participants 1:1 after screening to confirm stage 1 hypertension using home BP readings to either a control group receiving standard care lifestyle advice or an intervention group receiving standard care lifestyle advice plus an isometric exercise programme. Standard care advice consisted of verbal advice from the healthcare professionals conducting the visit and a leaflet containing NHS-approved advice (Supplementary file 1_Standard care advise leaflet). Participants included those over 18 years, with stage 1 hypertension, not on anti-hypertensive medication and without significant medical contraindications. The isometric exercise intervention consisted of a 6-month programme of static wall squats (Fig. 1), the intensity of which was tailored to the individual in an incremental isometric exercise test [33] delivered by a healthcare professional with intensity maintained using a measure device (bent and squat device, Fig. 1). Wall squats were conducted at home by participants in four bouts of 2 min with 2-min rests in between, three times a week [32]. All participants measured their blood pressure and heart rate at home at 4 weeks, 3 months and 6 months using a blood pressure monitor provided by the study. Participants carried out observed blood pressure measurements during baseline and remote follow-up appointments with a healthcare professional. Those in the intervention arm recorded their heart rate during exercise sessions to confirm intervention fidelity. Participants’ demographic and hypertension diagnosis details were recorded at baseline and were asked to complete a diet and exercise questionnaire at each assessment timepoint (baseline, 4 weeks, 3 months and 6 months). The study was conducted during the COVID-19 pandemic and adaptations to the design were required. Full details of the protocol can be found in Wiles et al. [32].

The aims of the embedded qualitative study were to determine the feasibility of delivering the isometric exercise; assess if healthcare professionals could deliver the intervention in an NHS setting; explore the willingness of healthcare professional stakeholders to consider IE as a treatment option for hypertension in routine practice; and understand participant experiences of undertaking isometric exercise and adherence [32].

### Participant recruitment and consent

Participants in the feasibility study intervention arm were contacted by email after 4 weeks of isometric exercise training to invite them to take part in an interview with a member of the research team as part of the qualitative study and provided with a full information sheet. Initially, it was intended to recruit participants to one of two focus groups to collect qualitative data; however, due to challenges with participant recruitment related to the COVID-19 pandemic, individual participant interviews were adopted to maximise data collection in the time available. Control participants that went on to accept the offer of an isometric exercise programme upon study completion were also approached by email, 4 weeks after the participant was given an isometric exercise programme.

### Healthcare stakeholder recruitment

Two groups of stakeholders were recruited by email containing a brief description and attached participant information sheet; firstly, healthcare professionals were recruited from NHS organisations not participating in the study through advertising amongst the research team’s networks in England. Prior to interview, they were provided with a summary of the feasibility study that included an introduction/rationale, participant eligibility criteria, details of primary and secondary objectives, study visits and assessments (Supplementary file 2_IsoFIT-BP Study Summary for HCPs). Secondly, healthcare professionals delivering the intervention at study sites were contacted by email to invite them to take part in the qualitative study towards the end of the study recruitment period. Interviews were conducted by the same researcher as participant interviews.

### Data collection

Interviews were conducted by a single research team member (RB, research manager) with experience undertaking interviews and qualitative research. Interviews were conducted using a semi-structured interview guide co-produced by two researcher team members and two public co-applicants. An initial list of questions was drawn up by the research team for participants which was then reviewed and refined by two research team members and two public co-applicants. The healthcare professional schedules were then posed similar questions but adapted to consider the view point of delivering the intervention rather than undertaking the intervention. The final interview schedules can be found as Supplementary files (Supplementary file 3_participant interview schedule, Supplementary file 4_Healthcare professionals at participating sites interview schedule and Supplementary file 5_Healthcare professionals at non-participating sites interview schedule). Interviews were scheduled at the participants’ convenience and consent was obtained using an online consent form (Qualtrics XM platform, https://www.qualtrics.com/uk/) for study participants and verbally for stakeholders prior to the interview. Interviews were held by telephone or online, audio-recorded and transcribed for analysis. Transcripts were not returned to participants for comment or correction. Qualitative data was collected in line with guidelines for using qualitative research in feasibility studies [34].

Between 27^th^ June 2022 and 15^th^ March 2023, *n* = 10 interviews were conducted with intervention arm participants (45% of total intervention group, *n* = 22) and *n* = 1 from the control group who opted for an exercise programme at end of study. Interviews lasted for between 30 and 60 minutes. The interviews aimed to draw out interviewees’ attitudes, feelings, beliefs and experiences regarding the intervention. In addition, *n* = 5 interviewees took part in four interviews (one interview had two participants from the same site) conducted between 26^th^ May 2021 and 20^th^ July 2021 with healthcare stakeholders at NHS sites not participating in the study. Interviews explored opinions on study design, attitudes towards the intervention itself and the willingness of the healthcare professionals to consider the isometric exercise intervention as a viable treatment option for patients, including barriers and facilitators for delivering and integrating this within their NHS care pathways for hypertension. Finally, *n* = 3 interviews were conducted with healthcare professionals responsible for delivering the isometric exercise intervention at NHS study sites (between 1^st^ April and 7^th^ September 2022). As well as covering similar elements explored in the non-participating site interviews, healthcare professionals delivering the intervention were asked to relay their experiences of delivering the intervention alongside relevant barriers and facilitators.

### Data analysis

All interview transcripts underwent inductive reflexive thematic analysis using Braun and Clarke’s [35] six-stage model. Drawing on Sweeney and colleagues [36] notion that the service user researcher unique perspective should be preserved rather than subsumed, the process involved multiple team members (including public co-applicants). The team independently read and re-read a subset of transcripts noting down initial codes (two team members reading across all transcripts). Transcripts were coded and data extracts collated by a single team member in a Microsoft office 365 Powerpoint application. Data collated included each code organised into sub-themes and higher-order themes. A collaborative meeting of all was then held to confirm, review, refine the final themes and ensure no new themes were identified. Participants will receive a summary of the findings upon publication of the results.

## Results

Eighteen interviews (*n* = 10 intervention arm participants, *n* = 5 healthcare professionals from non-participating sites and *n* = 3 healthcare professionals delivering the intervention) were analysed, with participant demographic characteristics shown in Table 1 indicating similarity across the qualitative study and overall cohort. Of the healthcare professionals interviewed, all those delivering the intervention were research nurses and those from non-participating sites were from mixed professional backgrounds including general practitioners (*n* = 2), pharmacists (*n* = 2) and a health technician (*n* = 1).

**Table 1:** Characteristics of study participants alongside embedded qualitative study participants

**Demographic information**	**Feasibility study participants**		**Embedded qualitative study participants**
	**Intervention (*n* = 22)**	**Control (*n* = 19)**	**Intervention arm only (*n* = 10)**
Age (years), mean (SD)	57.00 (15.15)	56.21 (14.34)	60 (11.93)
Age ≥ 50 years, *n* (%)	16 (72.7%)	14 (73.7%)	8 (80.0%)
Male, *n* (%)	9 (40.9%)	8 (42.1%)	3 (30.0%)
Ethnicity			
White	20 (90.9)%	17 (89.5%)	9 (90.0%)
Non-white	1 (4.5%)	1 (5.3%)	0 (0.0%)
Unknown	1 (4.5%)	1 (5.3%)	1 (10.0%)
Smoking status *n* (%)			
Smoker	2 (9.1%)	1 (5.3%)	1 (10.0%)
Previous smoker	6 (27.3%)	8 (42.1%)	3 (30.0%)
Non-smoker	14 (63.6%)	10 (52.6%)	6 (60.0%)

**Fig. 2:**
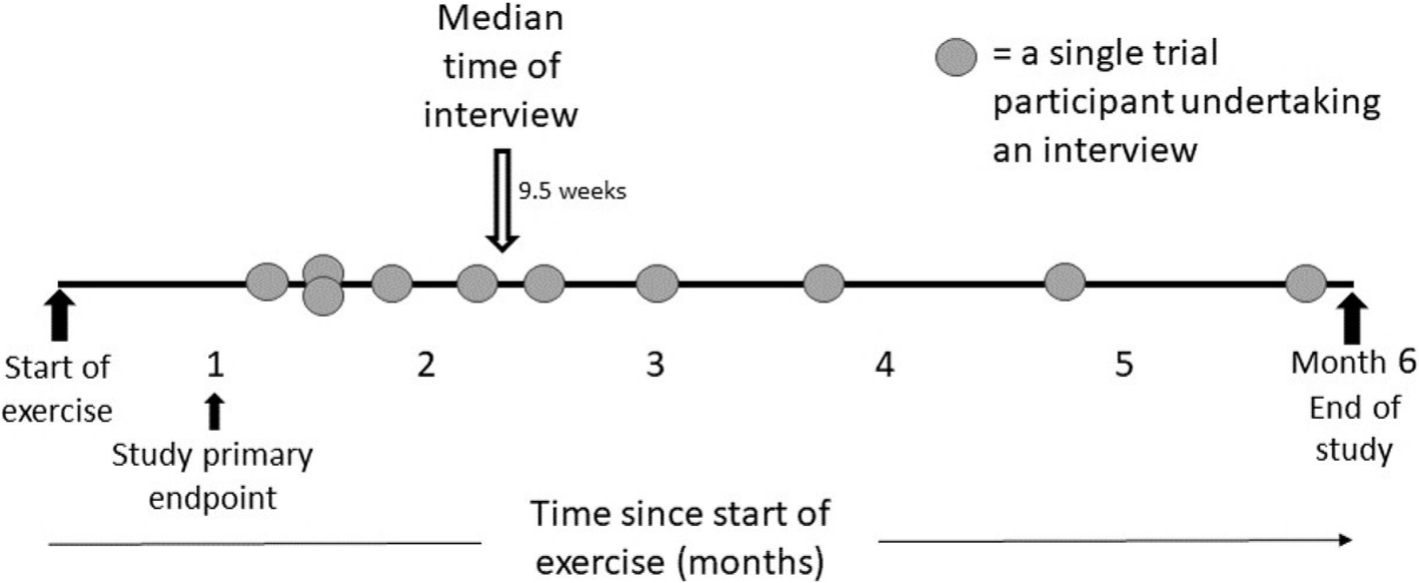
Study Interview timepoints

Timepoints of interviews during study delivery (Fig. 2) had a median of 9.5 weeks from the start of isometric exercise programme (range 5 weeks to 23 weeks) with the maximum number weeks of training per protocol being 24 weeks (6 months).

**Fig. 3:**
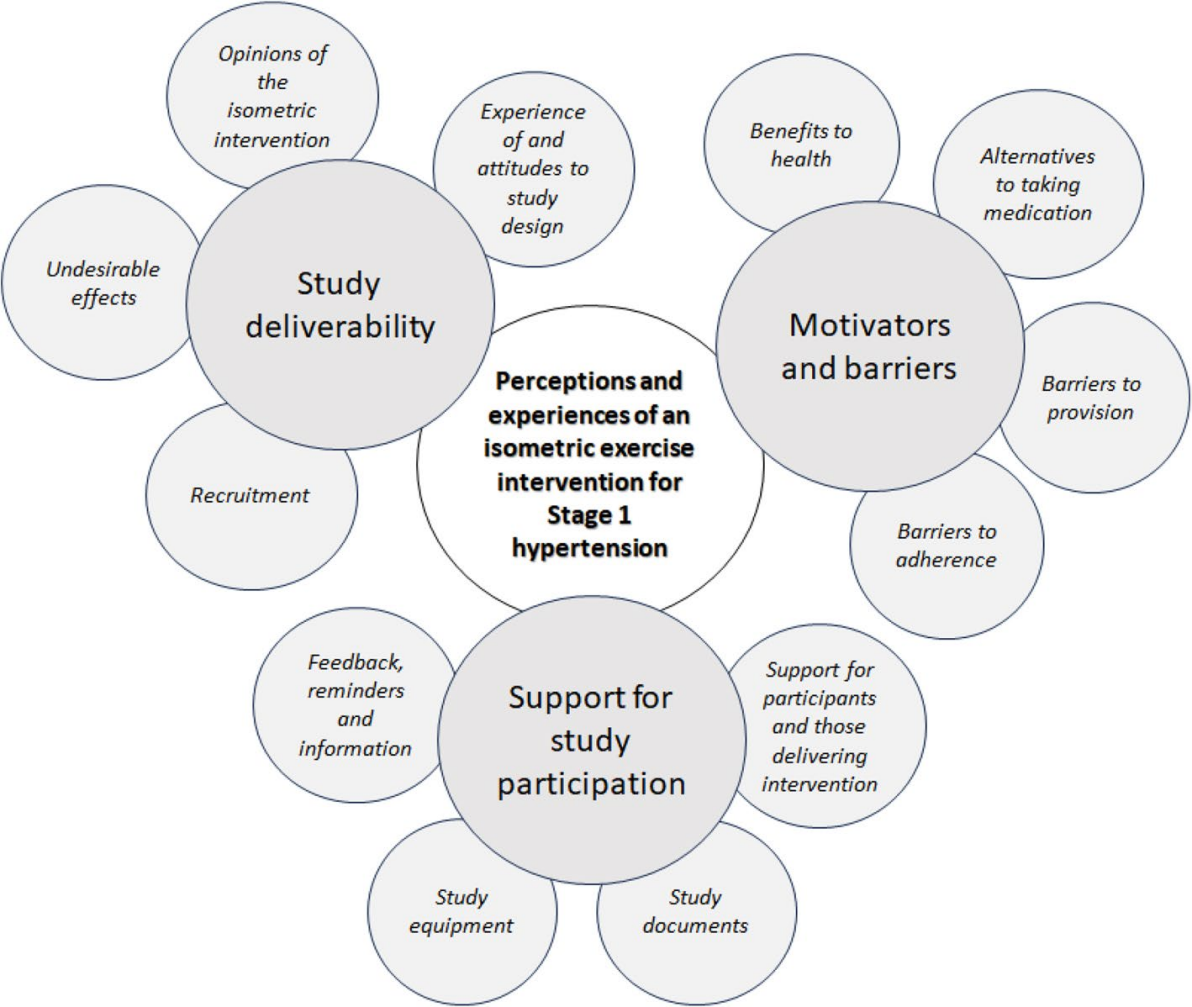
Summary of emerging themes and sub-themes

The emerging themes (Fig. 3) were as follows: (i) study design to enhance deliverability; (ii) motivation of participants and stakeholders; and (iii) optimum support for study participation.

### Study deliverability

Data provided rich insight into a number of key factors that supported or hindered study deliverability. Identifying and recruiting participants proved challenging, but once participants were identified, study sign up was straightforward. The study design was well received with a number of key considerations/revisions to improve deliverability identified. Most interviewees felt that isometric exercise was agreeable and easy to do. Some participants experienced transient muscle fatigue without effect on achieving their personalised programme. Some had concerns about the proper technique and welcomed reassurance by a healthcare professional in person to ensure correct performance of the exercise. Barriers included stakeholder concerns around accessibility of the exercise, particularly for those with musculoskeletal conditions limiting exercise ability, e.g. disability, knee or back problems. However, sample participants who experienced these problems did not find this a barrier. Interestingly, some participants experienced anxiety measuring their own blood pressure both alone and when being observed. White coat hypertension is defined as raised blood pressure in a clinic due to stress of this environment, leading to a higher blood pressure reading than would normally be experienced at home (with the term white coat referring to a healthcare professional). This finding that indicates white coat hypertension is a wide issue and also one that may be experienced when being observed remotely or through the stress of taking measurements alone at home without a healthcare professional present. This theme is organised into four subthemes below.

#### Mechanisms for recruitment

The method of recruitment for participants who agreed to be interviewed varied across the multi-modal recruitment strategies available from postal/email contact to eligible participants identified from clinical records, clinician direct referral, poster advertising at recruitment site/non-recruitment site locations (e.g. recruiting GP and hospital sites, pharmacies, supermarkets, research team organisations) or through to use of social media (Facebook). The most successful mode of recruitment was through social media (78% of 82 study participants recruited through this method). Most found that the study was easy to sign up to once they had become aware of the opportunity.

*‘…just press the button and see what comes of it …. I mean they made it very easy for me to fit into the programme.’ Participant 4.*

Organisational branding of the project within the information or advert seen by participants seemed to be important as recruits had a sense of contributing to goals in a positive way.

*‘…by doing this exercise I’m sure it’s helping the university and the hospital and all that sort of thing.’ Participant 3.*

Stakeholders at participating sites found the method of identifying eligible participants frustrating. This was due to the difficulty in searching primary care records accurately with reliable codes for stage 1 hypertension.

*‘So, the way the search was set up … it was bringing out a lot of patients that weren’t eligible.’ Healthcare professional 7.*

The study also exhibited a high screen failure rate (46%) which left some participants who experienced screen failure disappointed as they were keen to try the isometric exercise intervention and be included in the trial.

*‘…the people I have spoken to that unfortunately screen failed have been really, really keen to try this out.’ Healthcare professional 7*

#### Experience of and attitudes towards the study design

Stakeholders at non-participating sites felt that the study may not be targeting the optimum population for success and was not necessarily aligned to standard practice for hypertension in primary care where many stage 1 patients tend to be initiated on medication for hypertension immediately. Interestingly, this is in contradiction to first-line treatment for stage 1 hypertension which involves adaption of lifestyle factors in the absence of contra-indications [1]. Furthermore, it was felt that the study might be more attractive to those who were already exercising and *‘doing all the right things’* creating potential bias in inclusion.

*‘Yeah, so…usually we would say, at the point where we would diagnose hypertension, we would then refer on, but we’d probably also start them on medication at that point.’ Healthcare professional 3*

All participants interviewed expressed enjoyment in being part of the study and the study design was well received by all groups interviewed. The number of visits was overall acceptable to participants; however, stakeholders felt that the number of visits was too many in some cases.

*‘…the number of assessments and how regular contact is, is kind of about right, it’s not, I don’t end up dreading them, but oh for god’s sake now what do they want …’ Participant 6*

*‘I think the visits were fine and they all had direct contact with me if they needed to.’ Healthcare professional 7*

On-site visits being kept to a minimum was a positive element of the design as participants would rather not have to travel. However, stakeholders at participating sites preferred in-person contact. One participant found that conducting the isometric exercise in the comfort of their own home made it easier to do compared to when they tried it with the healthcare professional during the baseline visit. In some instances, the first try of the isometric exercise being conducted in person helped participants feel reassured that they were doing the exercise properly as they understood how it had to be done more easily through a physical visit compared to alone or online.

*‘I think going in person and for the initial, you know, where to measure the angle …, I think that if you’d have said, right sit against a wall and just YouTube video, I wouldn’t have been as precise about doing it correctly …’ Participant 6*

There was mixed opinion on the willingness of participants to be randomised. Most interview participants desired to receive the intervention, but were reassured that they could request to receive an isometric exercise programme at the end if allocated to the control group. Healthcare professionals delivering the intervention felt disappointment for participants allocated to the control arm, especially if they expressed a wish to try the isometric exercise.

*‘I just felt sorry for the control patients, particularly if they’re really excited at the prospect of doing the isometric exercise and then they don’t get it.’ Healthcare professional 6.*

#### Opinions of the isometric exercise intervention

Most participants liked the isometric exercise intervention and found it easy to do because it was short, did not make them exhausted and they did not sweat despite commonly experiencing some short-term muscular fatigue. There was some evidence that participants preferred the isometric exercise intervention as other types of lifestyle change were more difficult to do and stick to (e.g. losing weight through diet or reducing salt intake).

*‘Well, apart from my legs aching immediately afterwards, you know, within fifteen, twenty minutes, it’s almost as if I haven’t done it at all.’ Participant 10*

Some participants did find the isometric exercise difficult even if they considered themselves relatively fit; however, those that did were still happy to continue as it was short, not done every day and had a sense that it was potentially helping them. Some participants noted particular challenging elements of the exercise, including finding that a correct technique took time to achieve over the first week or so. Readily available advice was important alongside more explicit instructions (e.g. where to place their hands or what to do if their feet slip). These elements are detailed in theme 3, ‘Optimum support for study participation’. Some participants struggled with how they should rate their perceived exertion, which was a key data collection element that was difficult to judge and was felt to be subjective.

*‘The only problem that I found is that I tended to brace my arms on top of my thighs, and I was noticing that the heart rate after the fourth session was dropping …’ Participant 1.*

All healthcare professionals interviewed were positive about the intervention despite some not coming across this type of exercise before. They generally felt it was achievable for participants but with a need for clear evidence of efficacy for it to be implemented in routine practice. The need for evidence was echoed by participants.

*‘…if you can prove that it works then I think it’s … it’s a good treatment…’ Healthcare professional 2*

#### Undesirable effects

Stakeholders interviewed from non-participating sites were concerned about accessibility, in particular for those with mobility issues, chronic/acute pain or musculoskeletal problems. Despite this, no participants interviewed described any serious adverse effects other than transient muscle fatigue. One interviewee with knee problems did show concern over doing the isometric exercise programme and, although finding it slightly challenging due to discomfort, managed the exercise regardless.

*‘I do have, generally, a bit of a dodgy leg and knee and an ankle, but it hasn’t affected that.’ Participant 1*

Interestingly, some participants expressed anxiety over measuring their blood pressure, only doing it when they really needed to as they did not want to know or did not like the pressure of doing it. In addition, one participant expressed that they would have benefitted from being made aware that, during the study, they would have to read their blood pressure at home as the online observation by the healthcare professional caused particular anxiety. Despite this, participants expressed that getting used to taking their blood pressure whilst on study reduced their anxiety over time.

*‘Right, it’s made me feel a lot more comfortable about my blood pressure, it’s made me slightly less anxious about taking it at home …’ Participant 5*

### Motivators and barriers

Participants entered the study to benefit their own health and as a worthy option to improve their lifestyle. All participants interviewed expressed an unwillingness to take antihypertensive medication, wanting to try alternatives. All stakeholders, although positive about the intervention given effectiveness evidence, were concerned about how it might fit into standard practice. The intervention was felt to be complex and the training, although well received, needed to be refreshed regularly. Pieces of equipment needed were difficult to use by healthcare professionals and participants and space to do the exercise limiting. Most participants found a way to fit the exercise into their daily routines; however, there were reported issues where life events interrupted training. Despite these interruptions, most found a solution. Four sub-themes are now described:

#### Benefits to health

Many participants were motivated to take part in the research study due to the potential for a positive health outcome, feeling that the exercise was worth a try as they wanted to reduce their blood pressure. Many indicated altruistic motives, expressing a wish to help others in the future alongside a general interest in the study or helping to be part of seeing if the exercise was effective, key to its implementation.

*‘…so I know that blood pressure problems can cause a lot of, well, problems in life, especially as you get older, so it was mainly for my own benefit, to be honest.’ Participant 3*

There was a mixture of some participants feeling or knowing that they had seen an improvement and those that did not or thought their blood pressure had worsened. Having no sense of improvement whilst on study did not seem to affect motivation. Despite a sense of increased awareness of their blood pressure, participants did not convey a sense of overall difference in health. Healthcare professionals delivering the intervention did observe improvements in blood pressure in some instances.

*‘… it does look to be, I’d say it is, it is lower, it certainly looks better anyway …’ Participant 4*

Many participants were aware that they had high blood pressure or had been made aware by a healthcare professional, identified it themselves at home or through occupational health. Overall, being part of the study made people more aware of their lifestyle and how it related to blood pressure, likely due to receiving structured lifestyle advice as part of the study. Although some participants did not convey any changes to their lifestyle during the study, some felt more aware of sub-optimal elements of lifestyle resulting in minor or significant changes as a result. Importantly, those that were already active and exercised a lot found that it did not interfere with this and complemented existing routines. Struggling with weight issues was frequently cited and participants generally felt that they needed to do something about their weight.

*‘…it’s just opened my eyes to my health, my lifestyle, it’s making me take note of my blood pressure…’ Participant 2*

As part of their motivation, participants saw the study as a way of taking control of their health. Healthcare professionals supporting participants felt that the isometric exercise and study empowered individuals to manage their condition and influenced other lifestyle changes. All stakeholders interviewed felt that the intervention would be a good contribution to the ‘holistic’ offer for people with high blood pressure but particularly in combination with other things.

*‘I think that the people that are on it, some of them have massively allowed it to affect other aspects of their life, they’ve looked upon it as a starting point to try and better their overall health.’ Healthcare professional 6*

#### Alternatives to taking medication

All trial participants interviewed indicated that they were not keen to take medication for their high blood pressure, due side effects, the impact of medication on daily routines or wanting to try natural alternatives. Most stakeholders agreed and thought that many patients resist medication, leaving isometric exercise as an amenable option for those that wanted an alternative.

*‘…anything that means I don’t have to take pills, to me is of a benefit. I don’t like taking pills per se…’ Participant 10*

However, healthcare professionals interviewed also felt some patients preferred just to take simple medication as an option. The fact that preference for not taking medication was high in our sample points to possible bias of recruitment and that in future trials methods for recruiting people who are willing to take medication should be considered. Stakeholders interviewed from non-participating sites, in particular GP practices, had concerns about not prescribing medication, exacerbated by the need to reduce face-to-face appointments during the COVID-19 pandemic and the risks associated with not starting patients on anti-hypertensives despite the NICE guidelines for stage 1 hypertension as raised in our first theme [1].

*‘…they [GPs] might be hesitant to try this [without medication], because keeping your blood pressure high for months can have damaging effects…’ Healthcare professional 4*

#### Barriers to provision

There was significant concern amongst professionals interviewed about the ability to deliver the intervention as a service in a currently overstretched NHS climate. Furthermore, a few participants knew that their blood pressure was high but had not accessed services due to lack of understanding around the need to see a GP, the inability to access a GP or not being happy with their care. There were also concerns over how the intervention would fit into current practice, particularly given that primary care, where stage 1 hypertension care is provided, is stretched thinly alongside the worries about providing isometric exercise as an alternative to medication as mentioned above.

*‘…my blood pressure is a bit high, and I wasn’t happy with the response I was getting from my local clinic which was no response basically…’ Participant 4*

Professionals at non-participating sites were concerned that delivering the intervention would be complex and that proper training was critical to patients taking up the exercise, including the background and evidence for using isometric exercise to treat hypertension leading to a sense of it being out of their comfort zone. This was somewhat corroborated by healthcare professionals delivering the intervention as some found the technical elements of delivering the isometric exercise programme difficult at times (particularly use of equipment), needing project team support more than expected in order to feel confident. This may have been due to some having had longer than anticipated time between training and first participant recruitment, which meant they had forgotten some of the key training elements. It was also felt that the delivery process, with particular reference to the incremental isometric exercise test to determine optimum exercise intensity, could be simplified.

*‘I was terrified of learning how to do the isometric exercise test, because it did seem very complicated…’ Healthcare professional 6*

*‘But I think the process needs to be simplified with the teaching of the exercises … do they really need that specific angle for it to work or do they need just to be in a squat at the right angle that they can feel it …’ Healthcare professional 7*

Recruiting GP practice and hospital sites had minimal options to install additional equipment for the intervention delivery (wall charts) or exhibited a reluctance to do so.

*‘…at first, I thought will I have the space to do the IE test because that is a major problem that we do have, is hunting down space in which to see our patients.’ Healthcare professional 6*

#### Barriers to adherence

Both participants and stakeholders interviewed felt that the isometric exercise regime could easily fit into daily life. However, in some cases, life events meant that fitting in weekly sessions regularly was difficult, which created some non-adherence. There was also some indication of waning of commitment over time, sometimes due to personal stresses (e.g. being ill) and life events (e.g. going away) or getting towards the end of a week. Other situations that affected adherence included experiences of the COVID-19 pandemic such as catching COVID, holidays (particularly where equipment was forgotten or not taken along) and also family situations.

*‘… quite early on in the study, and it happened to coincide with me having a week’s holiday and about a week or two off, when I unfortunately contracted COVID…’ Participant 1*

Most who encountered issues which impacted their exercise regime managed to find a solution to elements that were getting in the way of adherence. In particular, the flexibility of when they could do the exercise, i.e. as long as it was three times a week, helped with adherence. Healthcare professionals supporting participants also provided instances where it did not fit into a participant’s lifestyle, particularly where this was due to work schedules, e.g. shift work. However, overall, there was a positive perception that the isometric exercise intervention was something that could be a lifetime intervention.

*‘…she works 12-hour shifts and then she’s got a long drive home afterwards and I think it was her lifestyle that she found difficult to stick to and she wasn’t particularly happy doing it over Christmas, but she did.’ Healthcare professional 7*

Linked to issues with available space in delivery sites, space to conduct and deliver the intervention was difficult in the home for some participants and created a barrier to both delivery and adherence. For participants, finding the right space in the house was important, including for the best conditions to do the exercise (e.g. a cool room) or to be able to keep it in one place. In some cases, this was hindered by limited wall space to do the exercise against.

*‘…we’ve just refurbished the whole house in the last year, so I don’t want to be marking walls and everything…’ Participant 4*

### Support for study participation

The study support provided to participants was well communicated and received. Healthcare professionals felt that participants welcomed positive support and that this was critical for adherence. Those delivering the intervention felt supported despite some lack of confidence in the early stages of delivering the exercise plans to participants. Participants had mixed views on whether they would use alternative methods for the study such as a digital application. Study documentation was highly commended in quality and information provided. One area for improvement was the order of the written diary with instructions not in a logical order. Accuracy of content and images was essential to enable participants to feel they were conducting the exercise properly. Some frustration was identified with the provided equipment both for conducting the exercise and recording study data. Feedback and reminders were deemed extremely helpful for participants supporting adherence alongside feedback at the end of the study being valuable. Text message reminders to promote adherence were helpful for some but a cause of frustration for others where they did not align with the days chosen to conduct the isometric exercise.

#### Support aimed at participants and those delivering the intervention

From the statements made, participants felt well supported whilst on study. There was awareness of the support available, and it was found to be helpful to know that support was there. When utilised, support was well received and commended with very few instances of negative support interactions with study team members. Only on one occasion was a negative experience expressed, which occurred when cover for a usual staff member was in place. This indicates the importance of dedicated study staff who know the study.

*‘…if I need to contact [for study problems], which I haven’t because there’s been no issues whatsoever, then I know someone’s at the end of the phone, either, or end of, by email.’ Participant 10*

In line with findings from the motivation theme, professionals expressed a need for positive support to ensure people continue with the programme, but it was felt in the current challenging NHS climate this contributed as a barrier within the other themes identified. Despite some lack of confidence of healthcare professionals delivering the exercise, all felt that they had enough support to overcome these difficulties to deliver the study.

*‘Some people need that encouragement and the checking in…’ Healthcare professional 6*

We asked participant interviewees if they would have preferred instructions in different formats, for example on a digital application (app) on a phone or tablet. Most participants did not feel that an app would improve their experience, and many did not think they would use one if available. However, some felt an app would be helpful, especially for those who were digitally competent and particularly useful when travelling away from home and to facilitate reminders. Those who were not keen on an app for delivery commented that they ‘*quite liked the idea of writing it down*’ (Participant 4), but some did not want to write it straight in the diary for neatness. There was a sense that older age groups in particular would find digital delivery of the programme a barrier, with some also commenting on a preference for a more personal touch.

*‘But I suppose if it was made on an app, it would notify you on the app that you needed to do it so it might be a bit more beneficial that way.’ Participant 2*

#### Study documents

There was considerable positivity towards the quality of the study documentation and instructions provided within the study. Participants felt that study documentation was easy to understand, easy to use, well written and not patronising. There were particular comments on the professional presentation of the diary as a bound document; however, the order of the diary appeared to be confusing with participants feeling surprised with something they had to do as they ‘turned the page’ as it was not in a natural order. There was also a sense that some documentation could be condensed where repetitive, particularly the diary and information sheets.

*‘Yes, I’ve had more than enough information. Sometimes maybe too much… This booklet I’m following… it seems to repeat a lot of things a few times…’ Participant 10*

Finally, one participant felt that the image in the diary was not accurate for the isometric exercise intervention leading to confusion that they were doing the exercise inaccurately with respect to placement of their feet at the end of the bend and squat measurement device.

#### Study equipment

The majority of participants found one or more pieces of study equipment difficult to use. Although this was not the case for the blood pressure measurement machines, it was commonly cited for the heart rate monitors and bend and squat measurement devices although less so for the latter. The main issues with the heart rate monitor equipment used to assess intervention fidelity included the following: their temperamental nature with it taking time to adjust to little quirks for use; a lack of instructions on how to use this piece of equipment; the monitor being hard to use or too big for certain body shapes (e.g. women or slender people); and that multiple changes of battery were sometimes needed during study which needed to be organised, taking time and effort to sort out.

*'The one thing that really nearly stopped me from getting started was the equipment, was being able to use the equipment efficiently … when I first got the equipment, I really had trouble with the using it …’ Participant 4*

Although the bend and squat device was thought to be well designed and helpful in reassuring participants that they were doing their wall squat to the right intensity, its eco-friendly cardboard composition was not perceived to be of good quality which led to frustrating experiences such as it falling away from the wall and being flimsy. One participant made the comment that this piece of equipment did not take into account the skirting board, which made them wonder if that might affect their isometric exercise intensity and as a result the effectiveness of the exercise.

*‘The cardboard measurement thing for the floor and wall is a dreadful piece of equipment’ Participant 3*

Those delivering the intervention to participants observed that in some cases the equipment was helpful, but in others it was a cause of frustration. Co-ordinating all the elements of the different pieces of equipment and doing the isometric exercise training at the same time was challenging for some.

*‘…at first it was sort of tricky to co-ordinate it with everything, you know, doing the watch and then hooking the strap round your bra strap, and then doing all that and then, you know, getting it all in line and doing it … It just took a while to sort of get into the rhythm if you like.’ Participant 3*

#### Feedback, reminders and information

Linked to the benefits for health and delivery theme above, feedback was deemed critically important for participants, who welcomed the encouragement it provided but indicated balance was required. The study provided text/email message reminders three times a week to do their exercise sessions, but there were mixed views on the helpfulness of these reminders. Some participants expressed that they found them very helpful whilst others found them annoying or frustrating, particularly when they did not match the days that they intended to do the exercise.

*‘I do get fed up with all the reminders coming through because they’re out of sync [laughs].’ Participant 5*

It was incredibly important to participants to have good feedback, advice and closure at the end of the study which included the wish to have advice for going forward after the study. Therefore, providing appropriate and sufficient feedback on their outcome at the end, including awareness of this as motivation to be part of the study, is important for future studies and may support adherence (theme 2).

*‘I do want to get some meaningful feedback out of this at the end of it as to what’s happened over the previous six months …’ Participant 4*

## Discussion

We have described the experiences and opinions of study participants and healthcare professional stakeholders (both delivering the study and those not involved in the study) of an isometric exercise wall squat programme for stage 1 hypertension within a randomised controlled feasibility study [32]. There are no other published randomised controlled trials of isometric wall squat for the treatment of hypertension in the NHS, despite laboratory-based studies indicating efficacy and secondary data analysis indicating superiority over other forms of isometric of exercise and other types of exercise programme (e.g. aerobic or combined aerobic/resistance training) [14]. The aims of the embedded qualitative study were to determine the feasibility of delivering the isometric exercise; assess if healthcare professionals could deliver and be willing to embed the intervention in an NHS setting; and understand participant experiences of undertaking isometric exercise and adherence. Qualitative evidence presented has informed the development of a follow-on effectiveness study and shares lessons for other relevant clinical trials. Although healthcare professionals were able to deliver the intervention, the evidence and feedback received through this embedded qualitative study has highlighted key barriers to uptake in standard practice. This has led to exploring simplified protocols for self-delivery of the same personalised wall squat programme [29, 37]. This development also reduces the need for equipment that was found to be sub-optimal by participants in this qualitative work. Finally, participants interviewed found the intervention acceptable and key ingredients for adherence identified. In a case study of isometric resistance training post-surgery for those with abdominal cancer, a qualitative study also found that isometric exercises are acceptable to patients [38]. Flexibility is key to enable participants to adapt and conduct the exercises within their comfort zone and fit the exercise into daily life notwithstanding certain life events having transient impact on adherence [38].

As this study was conducted during the COVID-19 pandemic, a number of adaptations were made that also contribute to learning points for efficient delivery of clinical trials including minimising in person visits, collection of data online and direct to patient recruitment [39]. Despite the majority of participants interviewed expressing ease in becoming involved in the trial through multiple recruitment routes, the most successful route of recruitment was through social media advertisement (Facebook). Baker et al. [40] shared learnings on use of social media for recruitment to an online monitoring eczema trial highlighting the significant reach of social media methods of recruitment, which also appeared to increase adherence. Despite this, social media advertisement did result in recruitment to particular participant groups (younger age groups) indicating that caution and a multi-modal approach to recruitment are essential to avoid lack of sample diversity [40]. A common issue in clinical trials is a lack of diversity of participants, despite social media advertising which in some studies has been shown to support diversity of recruitment [40]. As discussed by El-Galaly et al. [36], lack of diversity in clinical trials has considerable implications for impact and use in real-world settings. A key requirement in a follow-on effectiveness study is to achieve representative diversity for the population (in this case England) achievable through employing multiple recruitment strategies [41]. Involving a mixture of social media, in-clinic and outreach recruitment would likely allow more diverse recruitment and resolve issues in identifying participants from medical records not clearly coded for hypertension.

A finding of interest relevant to all clinical trials of hypertension was the anxiety participants experienced when measuring their blood pressure, including without anyone observing them. This anxiety may have led to falsely elevated readings whilst on study. Although it is important to control for white coat hypertension in clinical trials, Mahase [42] points to this condition itself being associated with a doubled risk of death from heart disease. Therefore, consideration of white coat hypertension inclusion in clinical trials may be important in addressing risk in this population.

Support arrangements and study documentation directed at both participants and healthcare professionals delivering the intervention were well received. There was mixed opinion from participants about the helpfulness of text message and/or email reminders which were sent three times a week. In a study by Panday et al. [43], text message reminders increased adherence to exercise in a clinical trial setting. Furthermore, personality type influenced the outcome of reminders with feeling types (who make decisions based on their personal value system and social considerations) showing higher adherence than thinking types (who rely heavily on objective information) and that emotional compared to logical messages were more effective [44]. One significant area for improvement was the design and support for equipment used in the study. Heart rate monitors were used to assess fidelity of the intervention by measuring peak heart rate achieved during exercise. Many participants struggled with this piece of equipment, expressing unsuitability for some body types, temperamental use and suboptimal battery life, the latter being noted in another study exploring the usability of cardiac monitors [45].

A significant motivator for taking part in the trial was a dis-inclination to take antihypertensive medication, mainly due to worries about side effects or wanting to try something natural or alternative. A systematic review of patient preferences for the management and treatment of hypertension supports this feature of our findings, with patients preferring treatment options with fewer comorbidities, faster responses, lower costs, more frequent blood pressure monitoring, easier administration and importantly fewer side effects as a significant consideration [46]. In this review, one study found that the majority of the participants believed that strengthening lifestyle changes would enable them to stop the medication, avoid additional medication or cure their hypertension alone [46]. These participants were optimistic about their ability to improve their lifestyle, attributing their expectations to scientific progress, good social and medical support, and stress relief [46]. A big driver for many participants was the potential benefit to their own health. Not only did taking part provide a structured exercise programme with the potential to lower blood pressure, but a potential placebo effect as a result of standard care advice in the control arm was observed. Such a placebo effect could significantly impact on the outcome of any trial with respect to blood pressure changes and attribution of effect to a lifestyle intervention.

Key barriers to the provision of and adherence to the isometric exercise intervention exist. In a systematic review conducted by Hall et al. [47], looking at the delivery of exercise interventions in primary care, key barriers reported included lack of time and training/guidelines as found in our study. In particular, professionals delivering the isometric exercise intervention felt that more training was required due to a lack of confidence and struggling with some items of equipment. Many doubted whether the intervention (as it stands) could fit into standard practice, and some commented that delivering an exercise intervention was out of the comfort zone of healthcare professionals. This is despite recommendations from the World Health Organization and National Institute for Health and Care Excellence that physical activity advice should be grounded in primary care [47]. Recruitment was also challenging due to overstretched services (even before this issue being exacerbated by the COVID-19 pandemic), with a shift to direct to patient advertising resulting in 78% of final recruitment. In another systematic review examining the effectiveness of physical activity interventions in primary care, delivery by a healthcare provider increased uptake; however, results were heterogeneous [48]. Therefore, given current demand, adaptation of structured exercise interventions for delivery direct to patients may be warranted.

A number of limitations should be considered alongside these results. Recruitment biases exist including self-selection of trial participants willing to have an interview, those willing to try an exercise intervention and those who did not want to take antihypertensive medication. Future trials could consider inclusion of those on antihypertensive medication, as lifestyle modifications are of benefit to all [7]. Study branding and social media use may have attracted those who related to the advertising. In addition, the majority of the qualitative sample was taken from the intervention arm. More interviews with control participants may have shed additional light on motivation to take part, the potential placebo effect and study experiences. Finally, our study sample had a significant lack of diversity.

## Conclusions

Implementing an isometric exercise intervention for stage 1 hypertension in the NHS is potentially feasible; however, definitive effectiveness evidence is critical. Patients liked this specific exercise intervention, and it helped them think about their overall lifestyle, giving them additional options for holistic treatment. Implementation will be challenging given current NHS demand and with the need for comprehensive training of healthcare professionals, unless alternative delivery options can be designed to mitigate these barriers.

### Supplementary Information

Supplementary Material 1

Supplementary Material 2

Supplementary Material 3

Supplementary Material 4

Supplementary Material 5

## Data Availability

Anonymous qualitative data is available on request to the corresponding author on reasonable request.

## Abbreviations

UK: United Kingdom of Great Britain and Northern Ireland
NHS: National Health Service
COVID-19: 2019 Novel SARS-CoV2 coronavirus
