# Supplementary material for: Experiences, acceptability and feasibility of an isometric exercise intervention for stage 1 hypertension: embedded qualitative study in a randomised controlled feasibility trial
Source: Pilot Feasibility Stud. 2024 Aug 26;10:113. doi: 10.1186/s40814-024-01539-8 (PMC11346254; doi:10.1186/s40814-024-01539-8)
Supplement: Supplementary file 2 — Supplementary Material 2 [file 40814_2024_1539_MOESM2_ESM.pdf]

Study Summary

IsoFIT-BP Study – Isometric exercise for people with raised blood pressure

Full Title of Project: Feasibility study to assess the delivery of a novel isometric exercise intervention for people with Stage 1 hypertension in the NHS

Background

As the most common long-term health condition in the UK and a primary risk factor for mortality, hypertension is a serious health problem. With every 20 mmHg increase in systolic BP above 115 mmHg and 10 mmHg increase in diastolic BP above 75 mmHg, the risk of death from cardiovascular disease doubles.

We have shown that a form of exercise, called isometric exercise (IE), can lower BP in people with both normal and high-normal (pre-hypertensive) BP and is a potential effective lifestyle intervention for hypertension. IE involves holding a fixed position for a period of time: muscles are used but there is no movement, e.g. leaning against a wall in a seated position. Only 24 minutes of IE a week are required to achieve reductions in BP in early studies, which can be easily carried out at home without costly equipment.

This study will determine the feasibility of delivering a personalised IE wall squat training programme to patients with Stage 1 hypertension (defined as a BP of 140-159/90-99 mmHg) for whom lifestyle changes would be recommended before treatment within a primary care NHS setting.

Study Aim and Objectives

Aim:

- To determine the feasibility of delivering a personalised isometric exercise intervention for people with Stage 1 hypertension in a primary care NHS setting.

Primary objectives:

- To assess if nurses/allied health professionals (e.g. health trainer, healthcare assistants, physiotherapists) can deliver isometric exercise prescriptions for Stage 1 hypertensive patients in a primary care NHS setting.
- BP data collected at baseline, 4 weeks, 3 and 6 months will be used to quantify the variance in BP change needed for calculating the sample size in a definitive randomised controlled trial.

Secondary objectives:

- Evidence the fidelity of the study intervention with respect to healthcare professional delivery and patient completion of IE.
- Determine short (4-week) and medium-term (3 and 6-month) adherence rates to IE intervention.
- Understand participant experiences of undertaking IE, adherence to the programme and continuation.
- Determine recruitment and attrition rates at recruiting GP sites to inform future trials (awareness of the possible negative effects of COVID-19 on recruitment rates).

- Explore the willingness of GPs, secondary care clinicians and healthcare professionals to consider IE as a treatment option for patients, including barriers and facilitators for delivering and integrating this within an NHS care pathway for hypertension.
- To establish the feasibility of cost of a full economic evaluation in a definitive trial.
- To investigate the feasibility of using observed home blood pressure readings for remote monitoring.

Eligibility Criteria

Inclusion criteria:

- Aged 18 or over
- Male or female
- Systolic BP 140-159
- Physically able to perform study intervention
- Participants able to give informed consent

Exclusion criteria:

- Currently taking anti-hypertensive medication
- Inability to provide informed consent
- If female, pregnancy or currently breast feeding
- Uncorrected congenital or inherited heart condition
- Previous history of any of the following:
  - myocardial infarction
  - moderate or severe stenotic or regurgitate heart valve disease
  - atrial or ventricular arrhythmia
  - coronary revascularization
  - stroke or Transient Ischaemic Attack
  - aneurysm
  - angina
  - other cardiovascular disease that, in the opinion of the investigator, would make the participant unsuitable for the study
- Estimated glomerular filtration rate <60 ml/min-1
- LVEF <=45%
- Participant included/enrolled in another clinical trial
- Medical condition that, in the opinion of the investigator, would make the participant unsuitable for the study

The Isometric Exercise programme

Participants will be randomly allocated to receive one of the following:

- Standard care / lifestyle advice alone for six months
- Standard care / lifestyle advice and an isometric exercise programme for six months.

Participants allocated to receive standard care advice and complete the isometric exercise programme will be asked to attend their GP practice for an exercise plan visit. This visit will be

conducted by a health care professional trained to administer the Incremental Isometric Exercise Test (IIET) e.g. a physiotherapist, nurse or health care assistant. During the visit they will be asked to complete the IIET which lasts approximately 10 minutes. This test is used to determine how hard they have to work during the exercise to get the most benefit. The test is split into two-minute continuous stages and over time the exercise gets harder. Participants begin the test at an easy exercise position, see figure A below. This position will be held for two minutes. After this, the exercise gradually gets harder by asking the participant to squat lower against the wall every two minutes until they reach the last stage (see figure E below) or until they feel they cannot hold the exercise position any longer. It is a continuous test so there are no rests between stages.

Using the results of this test, participants will then be given a personalised isometric exercise programme to do at home over the next six months. The exercise programme will involve doing the instructed wall squat exercise training session on three days each week at home. Each training session will consist of four bouts of wall squats for two minutes separated by two minutes of rest in between each round and will take approximately 15 minutes to complete in total. Full instructions will be given to participants in a training pack that they can take home. They will also be provided with a blood pressure monitor and a heart rate monitor with full instructions so that they can take home readings and record them in the study diary provided.

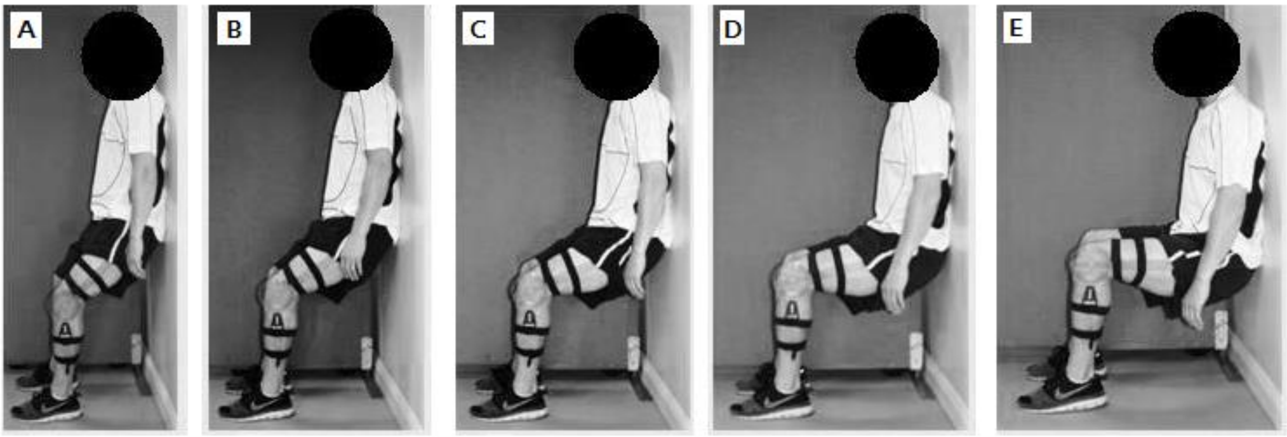

Study Flowchart

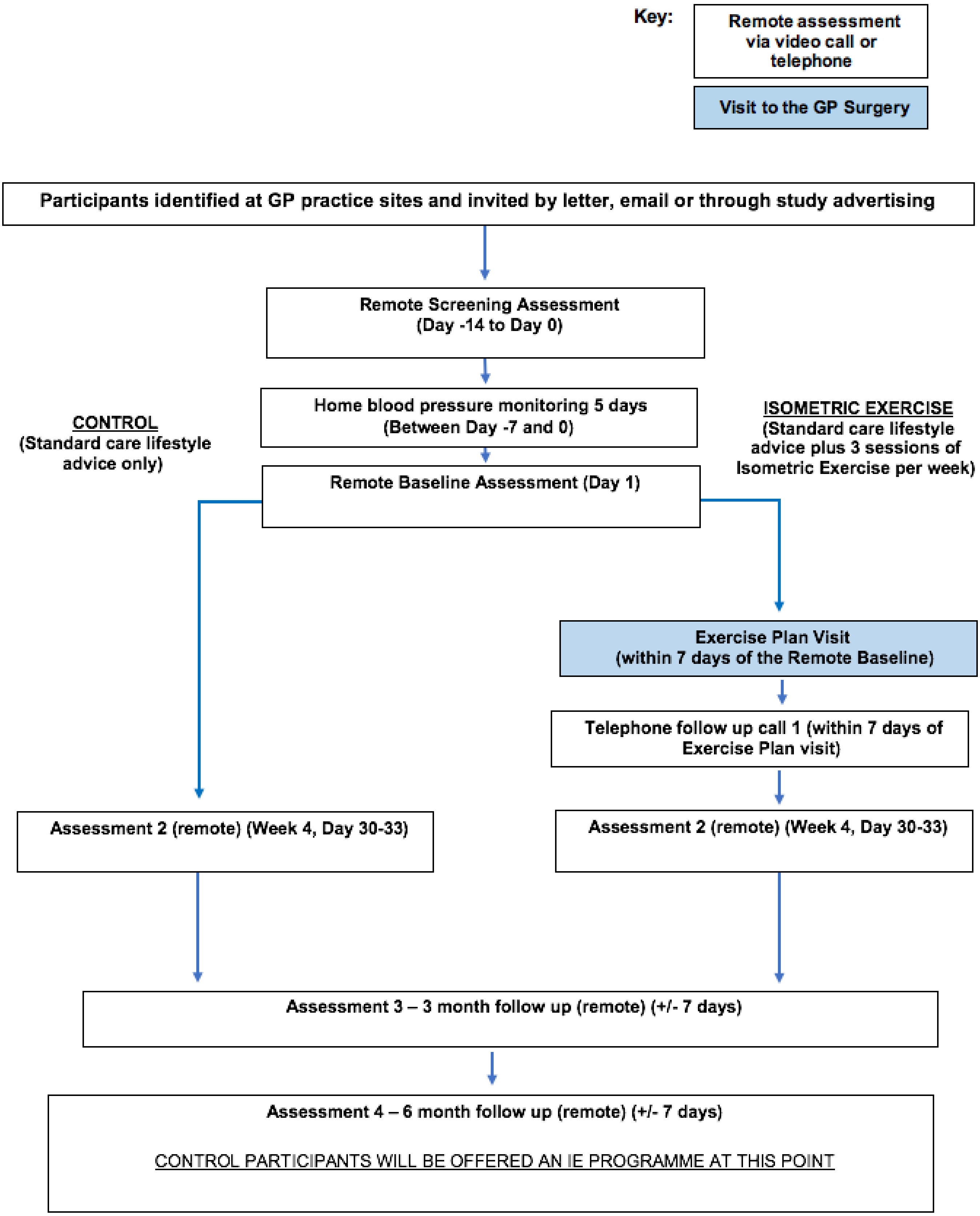

Schedule of Events

		Study time point					
**Assessment**	**Remote Screening Assessment Day -14**	**Baseline Assessment Day 1 (remotely)**	**Exercise Plan visit (within 7 days of Baseline)**	**Follow up Telephone call – IE arm only (within 7 days of IIET visit)**	**Assessment 2 Day 28-33 (Remotely)**	**Assessment 3 Month 3 (+/- 7 days) (Remotely)**	**Assessment 4 Month 6 (+/- 7 days) (Remotely)**
Medical history	X	X					
Concomitant Medication	X	X		X	X	X	X
Consent	X						
Observed blood pressure and heart rate		X			X	X	X
Isometric Exercise ability test	X						
Incremental Isometric Exercise test and Isometric Exercise programme provided (for those randomised to intervention arm only)			X				
Adverse Events review				X	X	X	X
Diet questionnaire		X			X	X	X
Exercise questionnaire		X			X	X	X
Quality of life questionnaire		X			X	X	X
Collection of Isometric Exercise diary (for those randomised to intervention arm only)				X	X	X	X
Collection of home blood pressure and heart rate readings	X (between Day -7 and Day 1)	X		X	X	X	X
Health resource use questionnaire					X	X	X
Isometric Exercise experience questionnaire					X		
