# Supplementary material for: Experiences, acceptability and feasibility of an isometric exercise intervention for stage 1 hypertension: embedded qualitative study in a randomised controlled feasibility trial
Source: Pilot Feasibility Stud. 2024 Aug 26;10:113. doi: 10.1186/s40814-024-01539-8 (PMC11346254; doi:10.1186/s40814-024-01539-8)
Supplement: Supplementary file 3 — Supplementary Material 3 [file 40814_2024_1539_MOESM3_ESM.pdf]

IsoFIT-BP – interview schedule – study participants

Pre-recording:

- Introduce myself
- Check that participant has read the information sheet
- Thank them for signing consent form/sort out signing the consent form
- Length of interview
- Confidentiality – although quotes may be used in study publications, they will not be identifiable in any published material
- Interview will be recorded, they can turn video off
- If they don't have an answer to a particular question, that's fine, we'll just move on
- Can stop the interview at any time
- Any questions before recording starts?

Interview questions:

1. **When did you start the isometric exercise programme?**

2. **How did you hear about the study?**

    Prompts if needed:
    - *Do you remember when you first became aware of the study?*
    - *Who first discussed the study with you?*

    With a follow on question

    i) What were your first thoughts about the study?

3. **What has been your experience of the isometric exercise programme so far?**

    Question prompts if required:

    - *Is the exercise programme easy to do? Do you have any problems with the isometric exercise programme and why?*
    - *Did you receive enough instruction or demonstration to be able to do the exercise at home?*
    - *Are you able to fit it into your lifestyle? Are you able to do the exercise at the same time and on same days?*
    - *Have you been given enough support – by the right sort of people, at the right time, in the right way? Were you able to get help if required?*
    - *Is there anything else that would have helped you with the exercise programme?*
    - *If this programme was provided in an app on your computer or phone or on the web, would you use it?*

4. **What impact has doing this exercise programme had on you?**

    Question prompts if required:

    - *Has the exercise programme lowered your BP?*
    - *If so, have there been any other specific changes you have noticed in your health?*

- *Has doing this exercise programme helped you to manage your blood pressure with more or less confidence?*
- *Has doing this programme impacted or changed other elements of your lifestyle or things that you do?*
- *If the programme has not changed anything, is there anything that might have been helpful?*
- *Compared to other possible treatments or interventions (e.g. drugs, other forms of exercise, diet changes) what do you think about this exercise programme?*
- *Is this something you will keep up in the long term? What made you keep doing, or not keep doing it?*
- *Did the amount of information about the exercise and your condition help assist you to manage it yourself?*

5. **What is your overall experience of taking part in the research study?**

Question prompts if required:

- *How did COVID-19 effect your willingness to take part in the study?*
- *What attracted you to take part in the study?*
- *What has kept you in the study?*
- *Was the information provided clear, particularly about what was involved in taking part in the study, your condition and the exercise programme?*
- *In the study, you were randomly assigned to receive the exercise programme with standard lifestyle advice or have standard lifestyle advice alone, were you happy to be randomly assigned to the groups in the study?*
- *Is there anything about the study you would like changed? E.g. number of visits, assessments etc.*

6. **We are now nearing the end of the interview, can I ask if there is anything we have not covered that you feel is important?**

7. **Just to finish: if you were in charge of this study, what one thing would you change?**

Thank the participant for their time and valuable contribution, and provide a point of contact in case they have any questions or queries in the future.

Tell the participant that we will arrange for their £15 shopping voucher to be sent to them via email.
