# Supplementary material for: Experiences, acceptability and feasibility of an isometric exercise intervention for stage 1 hypertension: embedded qualitative study in a randomised controlled feasibility trial
Source: Pilot Feasibility Stud. 2024 Aug 26;10:113. doi: 10.1186/s40814-024-01539-8 (PMC11346254; doi:10.1186/s40814-024-01539-8)
Supplement: Supplementary file 4 — Supplementary Material 4 [file 40814_2024_1539_MOESM4_ESM.pdf]

IsoFIT-BP – Interview schedule – healthcare professionals involved in delivering the study and intervention.

Pre-recording:

- Introduce myself
- Ask what their role is and clarify where they work
- Check that participant has read information sheet
- Length of interview
- Confidentiality – although quotes may be used in study publications, they will not be identifiable in any published material
- Interview will be recorded
- Tell them that I'll ask for consent to take part once recording has started
- If they don't have an answer to a particular question, that's fine, we'll just move on
- Can stop the interview at any time
- Any questions before recording starts?

Once recording has started:

- Ask for verbal consent to take part in the interview

Interview questions:

1. **How did you hear about the study?**

    Prompt if needed:
    - *Who first discussed the study with you?*

    With a follow on question

    i) **What were your first thoughts about the study?**

2. **What do you think about the isometric exercise programme?**

    Question prompts if required:

    - *Have you delivered the isometric exercise programme to participants and if so, how did it go?*
    - *What did you think about the programme initially when you started compared to now?*
    - *What do you feel are the good and bad elements of the exercise programme?*
    - *What do you feel the participants thought about the exercise programme and how did they do with it?*
    - *Do you think the intervention helped the participants to think about how they manage their condition successfully themselves?*
    - *Were there any elements of the isometric exercise programme that were difficult to deliver and why?*
    - *In delivering a programme like this, is there anything that might have helped or been helpful?*
    - *Did the intervention training provide you with the right information, resources and practical experience to deliver the exercise programme?*
    - *Were you given enough support to deliver the programme – was it by the right people, at the right time, done in the right way and contain the right information?*
    - *Is there any support you did not receive which you feel would have helped you?*
    - *Has taking part in this study changed your view of exercise interventions such as this?*

3. **Do you think it likely that an exercise intervention like this would become part of everyday practice?**

Question prompts if required:

- *Do you feel this intervention is something that could be used for people with hypertension?*
- *Balanced with your other responsibilities, what do you feel about this intervention and its feasibility?*
- *Compared with other possible treatments or interventions (e.g. drugs, other forms of exercise, diet changes) what do you think about this exercise programme?*
- *Has doing this programme impacted positively or negatively on the services you provide?*
- *Would you deliver this intervention for your patients?*
- *Do you feel it would be better if this programme was provided in an app on a computer or phone, or on the web?*

4. **What was your experience of taking part in the research study?**

Question prompts if required:

- *What attracted you to take part in the study and offer it to patients?*
- *Was the information about the study and training provided clear, particularly about what was involved in taking part in the study, how you would deliver the study and the exercise programme?*
- *What do you feel about the study design, e.g. recruitment approach, consent, screening, number of visits, assessments etc?*
- *Did you feel the randomisation for the study was straight forward?*
- *Was there anything about the study that was particularly difficult? E.g. number of visits, assessments etc.*

5. **We are now nearing the end of the interview, can I ask if there is anything we have not covered that you feel is important?**

6. **Just to finish: if you were in charge of this study, what one thing would you change?**

Thank the participant for their time and valuable contribution, and provide a point of contact in case they have any questions or queries in the future.
