# Supplementary material for: Experiences, acceptability and feasibility of an isometric exercise intervention for stage 1 hypertension: embedded qualitative study in a randomised controlled feasibility trial
Source: Pilot Feasibility Stud. 2024 Aug 26;10:113. doi: 10.1186/s40814-024-01539-8 (PMC11346254; doi:10.1186/s40814-024-01539-8)
Supplement: Supplementary file 5 — Supplementary Material 5 [file 40814_2024_1539_MOESM5_ESM.pdf]

IsoFIT-BP – Interview schedule – healthcare professionals from GP practices not involved in delivering the study and intervention.

Prior to the start of the interview, the interviewee will be asked if they have had a chance to read the information sheet and documents about the study provided. The interviewer will confirm that the interviewee's confidentiality will be maintained at all times and although quotes may be used in the study publications, they will not be identifiable in any published material. They will be asked if they have any questions before proceeding and to confirm verbally that they are happy to take part in the interview and that the interview will be recorded and used as described in the information sheet provided.

The following questions will be discussed with the interviewee:

1. **What do you think about the isometric exercise programme in this study?**

   Question prompts if required:

   - *What do you feel are the good and bad elements of an exercise programme such as this?*
   - *How do you think your patients might receive an exercise programme like this?*
   - *What sort of information, resources and/or practical experience might you need to deliver this exercise programme?*
   - *What support might you need in order to deliver a programme like this – from who, at what points and in what way?*
   - *What are your views on exercise interventions such as this?*

2. **Do you think it likely that an exercise intervention like this would become part of everyday practice?**

   Question prompts if required:

   - *Do you feel it is something that could be scaled up for use in primary care?*
   - *Balanced with your other responsibilities, what do you feel about this intervention and its feasibility?*
   - *Compared with other possible treatments or interventions (e.g. drugs, other forms of exercise, diet changes) what do you think about this exercise programme?*
   - *Are there any ways you feel delivering a programme like this may impact on your GP practice and the services you provide?*
   - *Would you deliver this intervention if given the choice and support to do so?*
   - *Do you feel providing exercise programmes like this would help you support patients with their health-related lifestyle?*

3. **Would you be willing to take part in a research study like this?**

   Question prompts if required:

   - *What might attract you to take part in a study like this and offer this to your patients?*
   - *What do you feel about the study design, e.g. recruitment approach, consent, screening, number of visits, assessments etc?*
   - *Do you feel there is anything patients will not be comfortable with or dislike about this study?*

4. **Can I ask if there is anything we have not covered that you feel is important?**

5. **To end, I would like to ask if you were in charge of this study, what one thing would you change?**

At the end of the interview, the interviewer will thank the interviewee for their time and valuable contribution. They will provide a point of contact in case any person taking part has any questions or queries in the future.
